# *GbMYBR1* from *Ginkgo biloba* represses phenylpropanoid biosynthesis and trichome development in *Arabidopsis*

**DOI:** 10.1007/s00425-020-03476-1

**Published:** 2020-09-29

**Authors:** Xiaojia Su, Yaying Xia, Wenbo Jiang, Guoan Shen, Yongzhen Pang

**Affiliations:** 1 Institute of Animal Science, Chinese Academy of Agricultural Sciences, Beijing, 100193 China; 2 Key Laboratory of Plant Resources and Beijing Botanical Garden, Institute of Botany, Chinese Academy of Sciences, Beijing, 100093 China; 3 University of Chinese Academy of Sciences, Beijing, 100049 China; 4 The Institute of Medicinal Plant Development, Beijing, 100193 China

**Keywords:** GbMYBR1, *Ginkgo biloba*, Phenylpropanoids, Repressor, Transcriptional regulation, Trichome

## Abstract

**Main Conclusion:**

GbMYBR1, a new type of R2R3-MYB repressor from *Ginkgo biloba*, displayed pleiotropic effects on plant growth, phenylpropanoid accumulation, by regulating multiple related genes at different levels.

**Abstract:**

*Ginkgo biloba* is a typical gymnosperm that has been thriving on earth for millions of years. MYB transcription factors (TFs) play important roles in diverse processes in plants. However, the role of MYBs remains largely unknown in *Ginkgo*. Here, an MYB TF gene from *Ginkgo*, designated as *GbMYBR1*, was found to act as a repressor in multiple processes. *GbMYBR1* was mainly expressed in the leaves of *Ginkgo.* Over-expression of *GbMYBR1* in *Arabidopsis thaliana* led to growth retardation, decreases in lignin content, reduced trichome density, and remarkable reduction in anthocyanin and flavonol contents in leaves*.* Proanthocyanidin content was decreased in the seeds of transgenic *Arabidopsis*, which led to light-brown seed color. Both qPCR and transcriptome sequencing analyses demonstrated that the transcript levels of multiple genes related to phenylpropanoid biosynthesis, trichome formation, and pathogen resistance were down-regulated in the transgenic *Arabidopsis.* In particular, we found that GbMYBR1 directly interacts with the bHLH cofactor GL3 as revealed by yeast two-hybrid assays. Our work indicated that *GbMYBR1* has pleiotropic effects on plant growth, phenylpropanoid accumulation, and trichome development, mediated by interaction with GL3 or direct suppression of key pathway genes. Thus, *GbMYBR1* represents a novel type of R2R3 MYB repressor.

**Electronic supplementary material:**

The online version of this article (10.1007/s00425-020-03476-1) contains supplementary material, which is available to authorized users.

## Introduction

The MYB transcription factors (TFs) comprise one of the largest families of transcriptional regulators in plants. They are involved in diverse processes, including development, stress responses, and metabolism (Ramsay and Glover 2005; Liu et al. 2015a; Roy 2016). Generally, these processes, including flavonoid biosynthesis, trichome initiation, mucilage production, and root hair initiation, are linked and regulated by a same set of TF complex. Among them, MYB-type TFs play key roles in the regulatory complex (Broun 2005).

In the model plant *Arabidopsis*, the ternary MBW (MYB-bHLH-WDR) complexes, which are composed of R2R3-MYB, bHLH (basic helix-loop-helix), and WD40 repeat (WDR) proteins, regulate the biosynthesis of flavonoids (e.g., anthocyanins and proanthocyanidins; Lepiniec et al. 2006; Li 2014). Anthocyanins production is transcriptionally activated when the WD protein (TTG1) and one of the bHLH proteins [TT8, Glabrous 3 (GL3), or Enhancer of Glabra 3 (EGL3)] interacts with one of the R2R3-MYB proteins such as PAP1 (Production of Anthocyanin Pigments 1), PAP2, MYB113, or MYB114 to form the MBW complex (Gou et al. 2011; Li 2014). TTG1 and TT8 can also interact with the R2R3-MYB-type TF TT2 to regulate proanthocyanidin accumulation in seeds (Gonzalez et al. 2009).

Meanwhile, MBW complexes also control epidermal cell patterning, including that of trichomes and root hairs (Lepiniec et al. 2006). One R2R3-MYB TF such as GL1 or Werewolf (WER), one bHLH protein such as GL3 or EGL3, and TTG1 compose the epidermal cell-specific MBW complexes (Payne et al. 2000; Li 2014). In addition to the MYBs in the MBW complex, individual MYBs in *Arabidopsis* (e.g*.,* MYB11, MYB12, and MYB111), could also activate the phenylpropanoid biosynthetic genes *CHS*, *CHI*, *F3H*, and *FLS* (Stracke et al. 2007). Thus, the regulation of flavonoid accumulation, trichome and root hair formation, and mucilage production were associated and sophisticated.

In addition to these positive regulators, several negative regulators are also involved in the regulation of flavonoid biosynthesis, including R3-MYB TFs (e.g., CPC and AtMYBL2) and R2R3 MYB TFs in the model plant *Arabidopsis*. CPC is a trichome-specific TF, which suppresses anthocyanin pathway genes by competing with PAP1/2 (Zhu et al. 2009). MYBL2 represses anthocyanin and proanthocyanidin pathways by interacting with the MBW complex (Dubos et al. 2008; Matsui et al. 2008). R2R3-type MYB TFs also act as repressors in the flavonoid pathway, which includes MYB3, MYB4, MYB5, MYB7, and MYB32 (Jin et al. 2000; Preston et al. 2004; Zhao et al. 2007; Fornale et al. 2014). Among them, MYB4 interacts with TT8 in MBW complexes and represses their transcriptional activities, and at the same time, MYB4 represses the expression of *MYB75*, *MYB90*, *TT2*, *ADT6,* and *CHS* genes (Jin et al. 2000; Wang et al. 2020). AtMYB5 regulates diverse developmental processes, including the regulation of flavonoids, seed mucilage, and trichome initiation in *Arabidopsis* (Li et al. 2009). Overall, the number of negative regulators is relatively few and their functions are lesser studied.

*Ginkgo* is a well-known medicinal plant with a valuable evolutionary history representative of gymnosperm plants. *Ginkgo* leaf extracts display multiple pharmaceutical functions (e.g., radical scavenging and antioxidant activities, anti-inflammation activity, and neuroprotective activity); therefore, *Ginkgo* has been utilized and investigated for centuries, leading to the identification of more than 60 different types of flavonoid compounds (van Beek and Montoro 2009; Liu et al. 2015b). Several genes associated with flavonoid biosynthesis have been identified and/or characterized in *Ginkgo* (Pang et al. 2005; Shen et al. 2006a, 2006b; Cheng et al. 2011; Hua et al. 2013; Xu et al. 2014a; Su et al. 2017; Zhang et al. 2018). Although a few R2R3-type MYB genes were identified via sequence analysis and two *MYB* genes were found to be related to flavonoid regulation (Xu et al. 2014a; Zhang et al. 2018), the regulatory mechanism of the flavonoid pathway by *MYBs* in *Ginkgo* remains unknown.

In the present study, we functionally characterized a highly expressed MYB TF gene from a previously constructed *Ginkgo* leaf transcriptome database (designated as *GbMYBR1*). *GbMYBR1* was highly expressed in leaves, and showed relatively high identity with several other R2R3-MYB TF genes from *Arabidopsis*. The over-expression of *GbMYBR1* in *Arabidopsis* repressed phenylpropanoid biosynthesis and trichome development, as well as the transcript levels of a set of phenotype-related genes. We found that GbMYBR1 physically interacts with the bHLH-type TF GL3 of *Arabidopsis*, as revealed in yeast two-hybrid assays. In addition, GbMYBR1 also repressed several regulatory genes as well as biosynthetic genes that are important for lignin and flavonoid accumulation, trichome development, and plant defense. Taken together, our data indicate that *GbMYBR1* is an evolutionarily unique R2R3 repressor gene that regulates multiple processes relevant to gymnosperm development.

## Materials and methods

### Plant materials

Roots, stems, and leaves of young seedlings, and fruits collected at different season of one *Ginkgo* tree grown in Beijing Botanical Garden, were collect and used in the present study as in our previous report (Su et al. 2017). The wild-type *Arabidopsis* accession Columbia (Col-0) was used for stable transformation, and the *Arabidopsis* plants were grown in a greenhouse with 16 h/8 h light/dark at 22 °C, and a relative humidity of 60%.

Seeds of transgenic and wild-type *Arabidopsis* lines were surface-sterilized with 20% NaClO and grown on half-strength MS medium. For the observation and analysis of anthocyanins, seeds were placed on half-strength MS medium containing 1% (w/v) sucrose under dark at 4 °C for 3 days for vernalization, and then transferred into a tissue culture room at 22 °C under 16 h/8 h light/dark (40 μmol photons m^−2^ s^−1^) for 14 d. These seedlings were then transferred onto half-strength MS medium containing 12% (w/v) sucrose and grown under 24 h light (80 μmol photons m^−2^ s^−1^) for 3 d. The 17-d-old seedlings were then collected for anthocyanin content and qPCR analyses.

The rosette leaves of the 21-d-old seedlings under normal growth condition were used for flavonol profiling and gene expression analyses. The main floral stem at the stage of 6.0 (Boyes et al. 2001) was used for trichome and gene expression analyses. The developing seeds of 4 d after pollination under normal growth condition were used for proanthocyanidin content and gene expression analyses.

### qPCR and transcriptome analyses

The extraction of total RNAs from various *Gingko* tissues was processed as previously described (Su et al. 2017). qPCRs were performed using the primer pair GbMYBR1-RT-F and GbMYBR1-R for *GbMYBR*1 gene (Table S1). *PP2A* gene was used as reference gene in qPCR analysis for *Arabidopsis* samples.

Total RNAs from seedlings, developing seeds, and the main floral stems of *Arabidopsis* plants were extracted using the TrizolA^+^ reagent (Tiangen, Beijing, China), and cDNAs were synthesized using reverse transcriptase with oligo primers (Promega, Hilden, Germany) after DNase I treatment. qPCRs were carried out with triplicates using SYBR Green reagent according to the manufacturer’s instructions (Kapa, Wilmington, USA). *PP2A* gene was used as house-keeping gene for the leaf and stem samples, and *UBQ-10* gene was used for the developing seed sample (4-d-old silique).

The aerial parts at the stage of 6.0 from both transgenic and wild-type *Arabidopsis* lines were collected and frozen in liquid nitrogen. Total RNAs were extracted using the RNA extraction kit (Promega), and total RNAs were sequenced with biological triplicates, using an Illumina Hiseq™ 2500 platform at Shanghai Hanyu Biotech Co. Ltd (Hanyu, Shanghai, China). Data assembling, annotation, and analyses were performed as previously described (Su et al. 2017). The expression levels of genes were calculated using the RPKM (reads per kb per million read) method. The genes with fold changes of more than 1.5-fold between the transgenic and wild-type *Arabidopsis* lines were selected. All the raw transcriptome data were deposited at the Sequence Read Archive of the National Center for Biotechnology Information (Accession number: SUB5848086).

### Sequence and phylogenetic analyses

Multiple sequence alignments of the deduced GbMYBR1 protein (Genbank Accession No. MH136603) and MYB proteins from other plant species were performed using DNAMAN software, and the neighbor-joining phylogenic tree was constructed using MEGA 6.0 with 1000 bootstrap replications (Tamura et al. 2013). Distance calculation was performed with Poisson correction and branch lengths were shown only when the values were above 50%. The GenBank accession numbers of all the MYB proteins are listed in Table S2.

### Generation of transgenic *Arabidopsis* plants

The open-reading frame of *GbMYBR1* gene (921 bp) was amplified using *pfx* high fidelity DNA polymerase, with cDNAs templates prepared from leaves and primers GbMYBR1-F and GbMYBR1-R (Table S1). The PCR condition was 94 °C for 5 min; 35 cycles of 94 °C for 20 s, 52 °C for 30 s and 68 °C for 60 s; followed by a final extension of 68 °C for 10 min. The *GbMYBR1* gene was cloned into the Gateway pENTR/SD/D-TOPO vector, and finally ligated into the pB2GW7 vector (Karimi et al. 2002) using LR reaction according to the manufacturer’s instructions (Invitrogen, Carlsbad, USA). The pB2GW7–GbMYBR1 plasmid was confirmed by sequencing, and then transformed into *Agribacterium tumefaciens* strain GV3101 for *Arabidopsis* transformation using the floral dipping method (Clough and Bent 1998). The seeds of T_0_ generation were plated onto the MS medium supplied with 10 mg L^−1^ phosphothricin for selection. The transgenic lines of T_1_ generation were confirmed by PCR and qPCR, and the homozygous lines of T_3_ generation were used for further analyses.

### Extraction and quantification of flavonoids

The 17-d-old seedlings under stress treatment were collected and ground into powder. Twenty milligrams of the powder were extracted with 500 μL methanol (0.1% HCl), cleaned with chloroform, and quantified at the wavelength of 530 nm. The anthocyanin content for the wild type was set as value of 100%, and the others were compared with that of the wild type.

The rosette leaves of 21-d-old seedlings and mature seeds were ground into powder in liquid nitrogen and then vacuum dried at − 40 °C for 24 h. The dried powder was used for flavonol analyses. Twenty milligrams of the powder were extracted with 600 μL 80% methanol, and 40 μL clean extract was analyzed on HPLC as previously described (Jiang et al. 2015). The main flavonol glycosides in *Arabidopsis* were determined according to the previous studies (Yonekura-Sakakibara et al. 2008; Yin et al. 2014).

Mature seeds of the transgenic and wild-type *Arabidopsis* were harvested, dried at 37 °C for 1 week, and ground into powder. Twenty milligrams of the powder were used for proanthocyanidin and flavonol analyses. The extractable proanthocyanidins and non-extractable proanthocyanidins were measured using DMACA (dimethylaminocinnamaldehyde) staining and butanol-HCl hydrolysis method, respectively, as previously reported (Pang et al. 2007).

### Trichome determination and histochemical staining assay of lignin

Trichomes on the rosette leaves and the main floral stem at the stage of 6.0 were numbered under microscope. Leaf area was calculated by its shadow on square paper of 1 mm^2^. Trichome density on the main floral stem was measured by the number of the trichome/the length of the corresponding stem.

The first floral stem was collected at the development stages of 6.0 from transgenic and wild-type plants, and the cross-sections were made with the first internode. For the visualization of lignified tissues, resin sectioning and toluidine blue staining were carried out as previously described (Lin et al. 2016). Resin sections (thickness 10 nm) were placed onto glass slides and dried on Slide Warmers at 60 °C. After the slices were dried, they were stained with toluidine blue staining for 1 min and gently washed by water and dried for observation under microscope.

### Yeast two-hybrid assay

The open-reading frame of *GbMYBR1* gene was cloned into both the pGADT7 vector with activating domain (AD) and the pGBKT7 vector with binding domain (BD), and the open-reading frames of regulatory genes of *Arabidopsis*: *TT2*, *TT8*, *TTG1*, *TTG2*, *GL3*, *EGL3*, *MYB12*, *MYB11*, *MYB111*, *MYBL2*, *PAP1*, *MYB113*, and *MYB114*, were cloned into the pGADT7 vector with activating domain. The plasmids were co-transformed into the yeast cell strain AH109. The yeast colonies grown on the synthetic SD-Trp-Leu dropout medium were confirmed by PCR. For interaction screening, the colonies were further transferred onto selective medium (SD-Trp-Leu-His-Ade) containing 2 mM 3-amino-1, 2, 4-triazole (3-AT), and 40 μg mL^−1^ X-α-Gal at 30 °C for 3–5 d.

### Pathogen infection assays

The monoclone of *Pseudomonas syringae* pv. tomato DC3000 (*Pst* DC3000) was used for inoculation of *Arabidopsis* leaves at a cell density of OD = 0.002 as previously described (Zeng and He 2010). Leaves of 4-week-old plants were inoculated with *Pst* DC3000 dilution by infiltration with a blunt-end syringe, and MgCl_2_ solution (10 mM) was used as mock control. *Arabidopsis* plants were covered under sealed hood for 24 h after infiltration, and they were then transferred to normal growth condition afterwards for 4 d.

Spores of *Botrytis cinerea* strain 2000 were used for inoculation of *Arabidopsis* leaves at a concentration of 2 × 10^5^ spores mL^−1^ as previously described (Sham et al. 2014). Spores diluted in 2% glucose/glycerol (4:1) were sprayed on 5-week-old *Arabidopsis*, and glucose solution (2%) was used as mock control. *Arabidopsis* plants were covered under sealed hoods and kept under dark 24 h after spray inoculation, and they were transferred to normal growth condition afterwards for 4 d.

Accumulation of hydrogen peroxide was detected as an indicator of damage by pathogen inoculation as previously reported (Niu et al. 2010). Inoculated *Arabidopsis* leaves were stained in DAB solution (1 mg mL^−1^ diaminobenzidine with 50 mM ascorbic acid, pH 3.8) under light for 8 h at room temperature. Subsequently, stained leaves were cleared with 95% ethanol and kept in 50% ethanol for visualization under light microscope.

## Results

### Isolation and sequence analysis of *GbMYBR1*

**Fig. 1:**
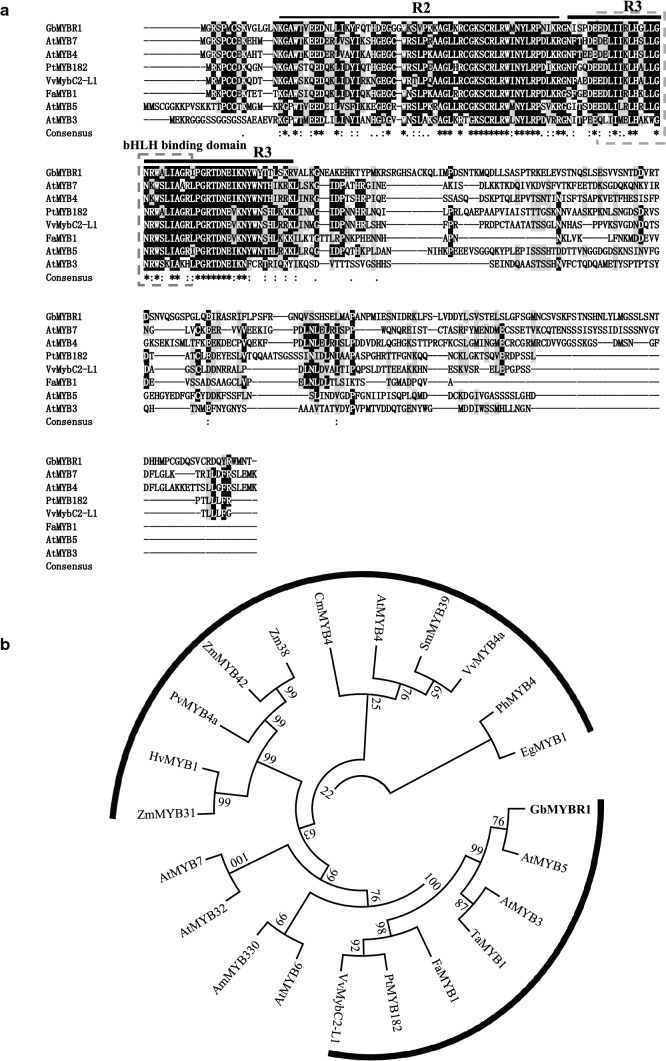
Sequence and phylogenetic analyses of the deduced GbMYBR1 with other functionally characterized R2R3-MYB TFs. **a** Multiple sequence alignment of the amino acid sequences of GbMYBR1 and other R2R3-MYB TFs. The R2 and R3-MYB domains are indicated with solid black lines and the bHLH-binding domain is marked with dashed box. White letters on a black background represent residues that are identical. Black letters on a dark gray background indicate highly conservative changes over 75% and black letters on a light gray background indicate conservative changes over 50%. **b** Phylogenetic analyses of GbMYBR1 and functionally characterized R2R3-MYB TFs from other plant species. The phylogenetic tree was constructed using Clustal W for multiple sequences alignments and a neighbor-joining tree with 1000 replicates was constructed using Mega 6.0. GenBank accession number for protein sequence is listed in Table S2

In a previous study, we developed a *Ginkgo* leaf transcriptome database (Su et al. 2017), and found that one of the ESTs for an MYB TF gene was highly represented. Moreover, this EST had a complete open-reading frame and its expression level was highly related to flavonoid accumulation in leaves (Fig. S1). To further characterize the function of this MYB TF gene, its open-reading frame (designated as *GbMYBR1*) was isolated with cDNA prepared from *Ginkgo* leaves. The *GbMYBR1* gene encoded a protein of 307 amino acid in length, which shows relatively low identity (approximately 30%) with MYB3, MYB4, MYB5, and MYB7 from *Arabidopsis*, PtMYB182 from *Populus*, FaMYB1 from *Fragaria* × *ananasa*, and MybC2-L1 from *Vitis vinifera* (Fig. 1a). In addition, the deduced GbMYBR1 protein showed less than 25% identity to *Ginkgo* MYBF2 that was reported to suppress flavonol and anthocyanin biosynthesis in *Arabidopsis* (Xu et al. 2014a).

Sequence alignment showed that the deduced GbMYBR1 protein has a highly conserved R2R3 DNA-binding domains at the N-terminus (Fig. 1a), while the C-terminal domain is more divergent both in sequence and in length. A R/B-like bHLH-binding motif ([D/E]Lx2[R/K]x3Lx6Lx3R) as previously reported (Zimmermann et al. 1997), was also present in the R3-DNA-binding domain of GbMYBR1 (Fig. 1a).

In a phylogenetic tree consisting of GbMYBR1 and several MYB repressors related to phenylpropanoid pathway from various plants, different MYB repressors were grouped into distinct clades (Fig. 1b). Specifically, these MYB TFs were grouped into two major clades, the MYB4 clade and the MYB5 clade. GbMYBR1 was positioned in the clade containing AtMYB5, AtMYB3, TaMYB1, FaMYB1, VvMybC2-L1, and PtMYB182. GbMYBR1 was clearly separated from AtMYB4 despite their relatively high identity (Fig. 1b). The distinct position of GbMYBR1 in the phylogenetic tree may be attributed to the high divergences in its C-terminal region.

### Expression profiles of *GbMYBR1* gene

**Fig. 2:**
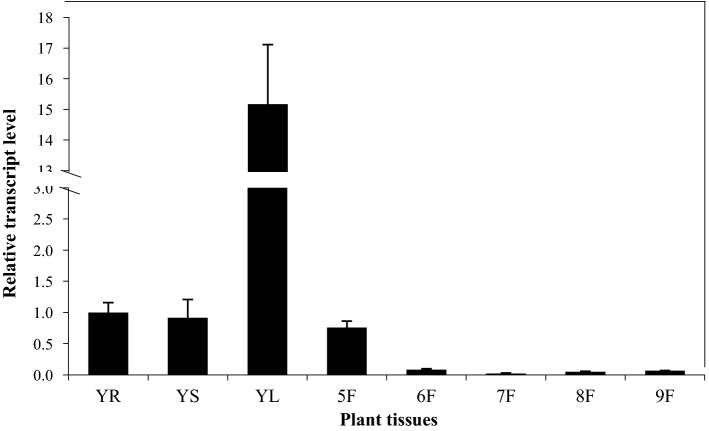
The relative transcript level of *GbMYBR1* in different tissues of *Ginkgo*. The relative transcript levels of *GbMYBR1* in various tissues and fruits at different months as detected by qPCR. YR, young root; YS, young stem; YL, young leaves; 5F-9F, the fruits of May–September. The transcript level of *GbMYBR1* in the young roots was set as value of 1.0. Values show the means and standard deviations of triplicates

To test the tissue-specific expression pattern of *GbMYBR1* in *Ginkgo*, we performed qPCR using RNAs derived from roots, stems, leaves of the young seedlings, and the fruits/seeds at different developmental stages (5F-9F, fruits developed from May to September). It was revealed that *GbMYBR1* was mainly expressed in young leaves, and the expression level of *GbMYBR1* in young leaves was more than 12-fold higher than the levels in roots or stems (Fig. 2). In addition, *GbMYBR1* was also expressed in fruits, and the relative transcript level of *GbMYBR1* decreased as fruit matured (Fig. 2). Overall, the relative transcript level of *GbMYBR1* was significantly higher in leaves than in fruits (Fig. 2), indicating that *GbMYBR1* mainly functions in *Ginkgo* leaves.

### Ectopic over-expression of *GbMYBR1* gene negatively impacts *Arabidopsis* growth

**Fig. 3:**
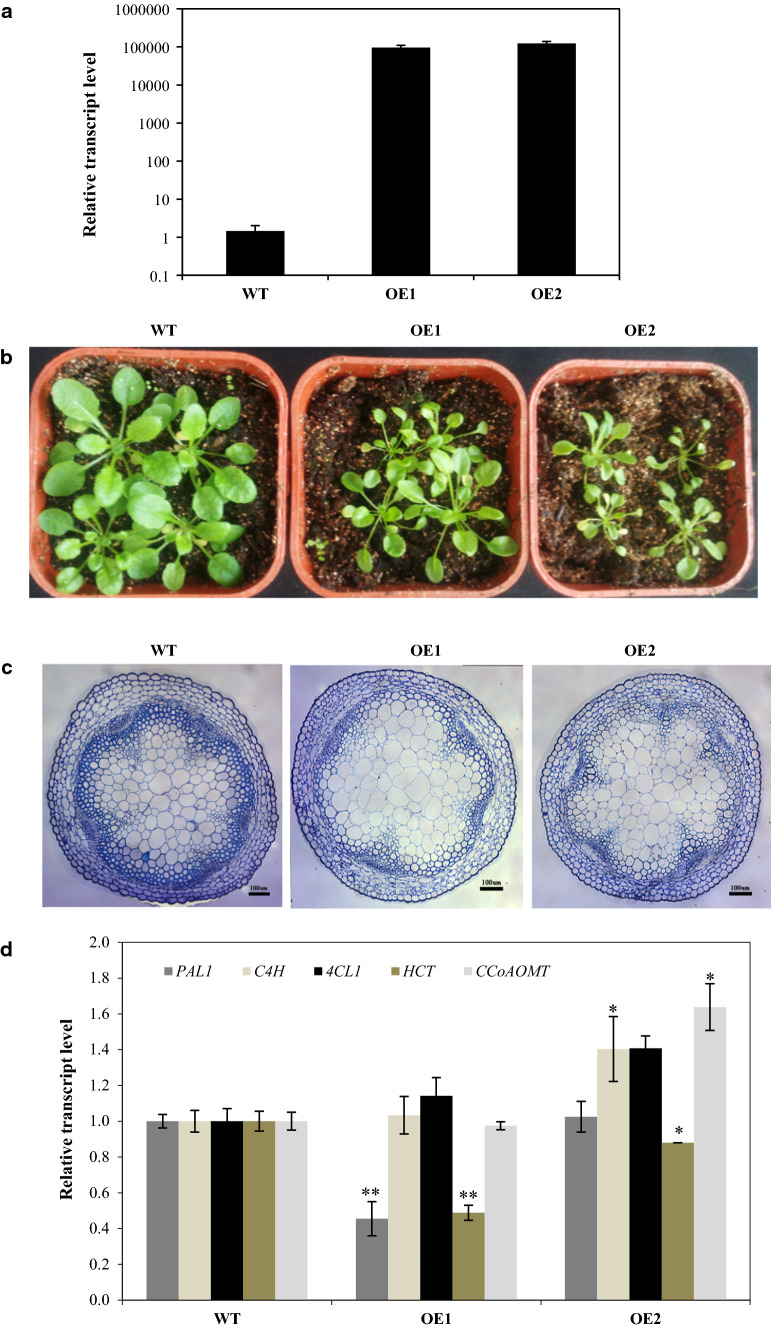
Phenotype of the transgenic *Arabidopsis* seedlings over-expressing *GbMYBR1*. **a** The relative transcript levels of *GbMYBR1* in the seedlings of transgenic *Arabidopsis* lines as detected by qPCR, *PP2A* was used as house-keeping gene. The transcript level of the wild type was set as value of 1.0. **b** Phenotype of the transgenic seedlings (OE1 and OE2) compared with the wild type (WT). **c** Cross section of the first internodes of the transgenic plants (OE1 and OE2) and the wild type stained with toluidine blue. **d** The relative transcript levels of genes involved in lignin pathway were detected by qPCR. Data are presented as mean ± SD. Asterisks denote *t* test significance: *P < 0.05, **P < 0.01, *n* = 3

*GbMYBR1* was over-expressed in the model plant *Arabidopsis* for in vivo functional characterization, as no successful transformation has yet been reported in *Ginkgo*. The expression level of *GbMYBR1* in six transgenic *Arabidopsis* lines was determined by both RT-PCR and qPCR, and two transgenic lines with obvious phenotypes and relatively high transcript levels were used for further analyses (Fig. 3a).

The transgenic *Arabidopsis* plants over-expressing *GbMYBR1* showed obvious growth retardation as compared to the wild type under normal growth conditions (Fig. 3b), which may be due to the effect of *GbMYBR1* on lignin content. Because, normally, when lignin is reduced, plant growth is negatively affected. We thus measured lignin content using toluidine blue staining in the over-expression (OE) lines along with wild type. It showed that the blue staining (indicative of lignified cell walls) was weaker in the transgenic lines than in the wild type, indicating that lignification was suppressed in the OE lines (Fig. 3c).

The effect of *GbMYBR1* on the monolignol biosynthetic pathway was confirmed by qPCR analysis. Only *HCT* gene was significantly decreased in both tested OE lines as compared to the wild type, among the other lignin pathway genes that were tested by qPCR including *PAL*, *C4H*, *4CL*, and *COMT* (Fig. 3d). This down-regulation of *HCT* is consistent with the decrease of lignin staining in the transgenic lines, as *HCT* encodes the enzyme that catalyzes the rate-limiting step of the lignin pathway in *Arabidopsis* (Besseau et al. 2007).

### Ectopic over-expression of *GbMYBR1* gene down-regulates flavonoid biosynthesis in the leaves of *Arabidopsis*

**Fig. 4:**
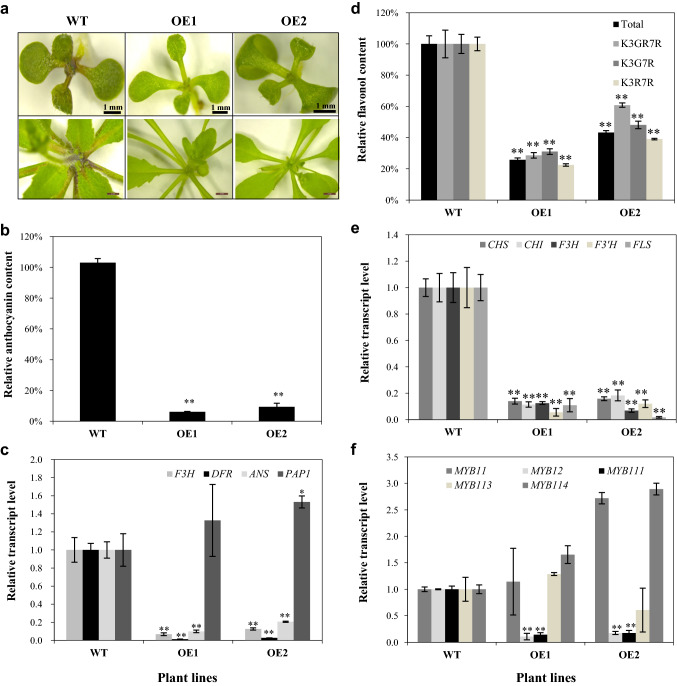
Anthocyanin and flavonol accumulation in the transgenic *Arabidopsis* over-expressing *GbMYBR1* and the wild-type lines. **a** Anthocyanin accumulation in 17-d-old seedlings (upper panels) and the mature *Arabidopsis* plants (lower panels). **b** The relative anthocyanins content in the 17-d-old seedlings of the transgenic *Arabidopsis* and the wild type. The anthocyanin level in the wild type was set as a value of 100%. **c** The relative transcript levels of genes involved in the anthocyanin sub-pathway were determined by qPCR. The transcript level of individual gene in the wild type was set as value of 1.0, respectively. **d** The relative flavonol content in the 21-d-old seedlings of the transgenic *Arabidopsis* and the wild type. The flavonol level in the wild type was set as a value of 100%. **e**–**f** The relative transcript levels of genes involved in the flavonol sub-pathway were determined by qPCR. The transcript level of individual gene in the wild type was set as value of 1.0, respectively. Data in **b**–**f** are presented as mean ± SD, Student’s *t* test (*n* = 3, *P < 0.05, **P < 0.01)

Under stress condition (e.g., high sucrose and light), purple pigmentation was obvious in the wild-type *Arabidopsis* seedlings (Fig. 4a, left upper panel), but no obvious purple pigmentation was observed in the two OE lines (Fig. 4a, upper middle and right panels). Dramatic reduction in anthocyanin content (about 90%) was found in the seedlings of the two OE lines in comparison to the wild type (Fig. 4b). Even in mature plants, no obvious anthocyanin accumulation was observed in the rosette leaves or main stem of the OE lines (Fig. 4a, lower panels), implying that anthocyanin accumulation was severely suppressed by *GbMYBR1* in the transgenic *Arabidopsis*.

To dissect potential mechanism for anthocyanin reduction, qPCR was performed to determine the transcript abundance of several anthocyanin pathway genes. The relative transcript levels of three key biosynthetic genes *F3H*, *DFR*, and *ANS* were significantly reduced in the seedlings of OE lines relative to the wild type (Fig. 4c). However, the transcript level of *PAP1*, a master regulatory gene of the anthocyanin pathway, did not change in the two transgenic lines (Fig. 4c). These results indicate that *GbMYBR1* affects anthocyanin content by reducing the transcript level of *DFR*, *F3H*, and *ANS* genes.

Flavonols are the major flavonoid compounds present in *Arabidopsis* seedlings, in particular the three major flavonol glycosides K3RG7R (kaempferol 3-O-(rhamnosyl(1amnglucoside)-7-O-rhamnoside), K3G7R (kaempferol 3-O-glucoside-7-O-rhamnoside), and K3R7R (kaempferol 3-O-rhamnoside-7-*O*-rhamnoside) (Yonekura-Sakakibara et al. 2008; Yin et al. 2014) (Fig. S2). HPLC analysis revealed that these three compounds were significantly decreased in the two OE lines (Fig. 4d, Fig. S2). Consequently, total flavonol contents were decreased by more than 50% in seedlings of the two OE lines compared to wild-type seedlings (Fig. 4d). qPCR analyses showed that several biosynthetic genes (e.g., *CHS*, *CHI*, *F3H*, *F3′H*, and *FLS*) and regulatory genes (e.g., *MYB12* and *MYB111*) were significantly decreased in the seedlings of the two OE lines (Fig. 4e, f). These results indicate that *GbMYBR1* negatively regulates flavonol accumulation in *Arabidopsis* seedlings through the down-regulation of biosynthetic genes as well as key regulatory genes in the flavonol pathway.

### *GbMYBR1* over-expression reduced trichome density in *Arabidopsis*

**Fig. 5:**
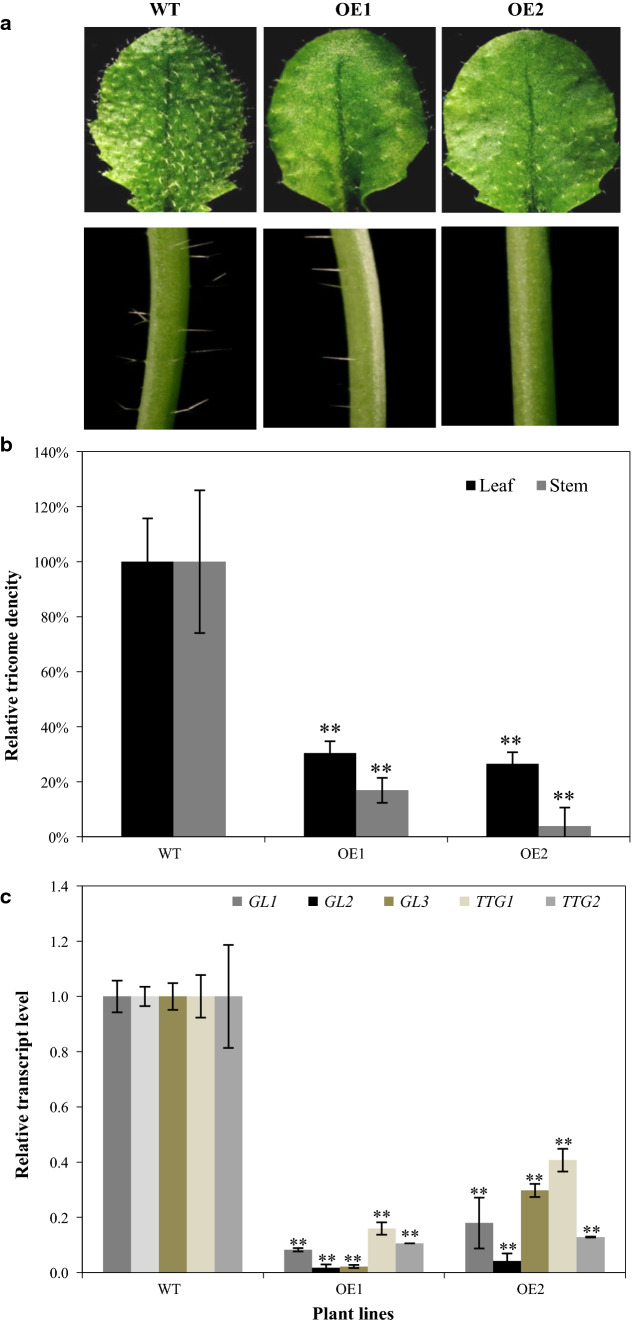
The trichome phenotype of transgenic *Arabidopsis* over-expressing *GbMYBR1* as compared to the wild type. **a** Trichome phenotypes of the transgenic lines (OE1 and OE2) compared with wild type in rosette leaves (upper panel) and the base of the main floral stem (lower panel). **b** Relative trichome density in rosette leaves and the base of main floral stem. The trichome density in the wild type was set as a value of 100%. **c** The relative transcript levels of genes involved in trichome development as determined by qPCR, and those in the wild type were set as value of 1.0, respectively. Data in **b-c** are presented as mean ± SD, Student’s *t* test (*n* = 3, *P < 0.05, **P < 0.01)

We found that trichome density on both the rosette leaves and the main floral stem was significantly decreased in the transgenic lines (Fig. 5a, b), but no significant differences were found in length or branch number of trichomes (Fig. S3a). In addition, no obvious differences in root hair or seed mucilage accumulation were observed between the transgenic and the wild-type lines (Fig. S3b, c).

To unravel the potential molecular mechanism involved in trichome development, qPCR was carried out to determine the transcript levels of regulatory genes related to trichome development. The relative transcript levels of *GL1*, *GL2*, *GL3*, *TTG1*, and *TTG2*, were all significantly decreased in the transgenic lines relative to wild type (Fig. 5c). Taken together, these results suggest that *GbMYBR1* negatively regulates trichome development by down-regulating regulatory genes.

### *GbMYBR1* over-expression affects proanthocyanidin and flavonol biosynthesis in seeds of *Arabidopsis*

**Fig. 6:**
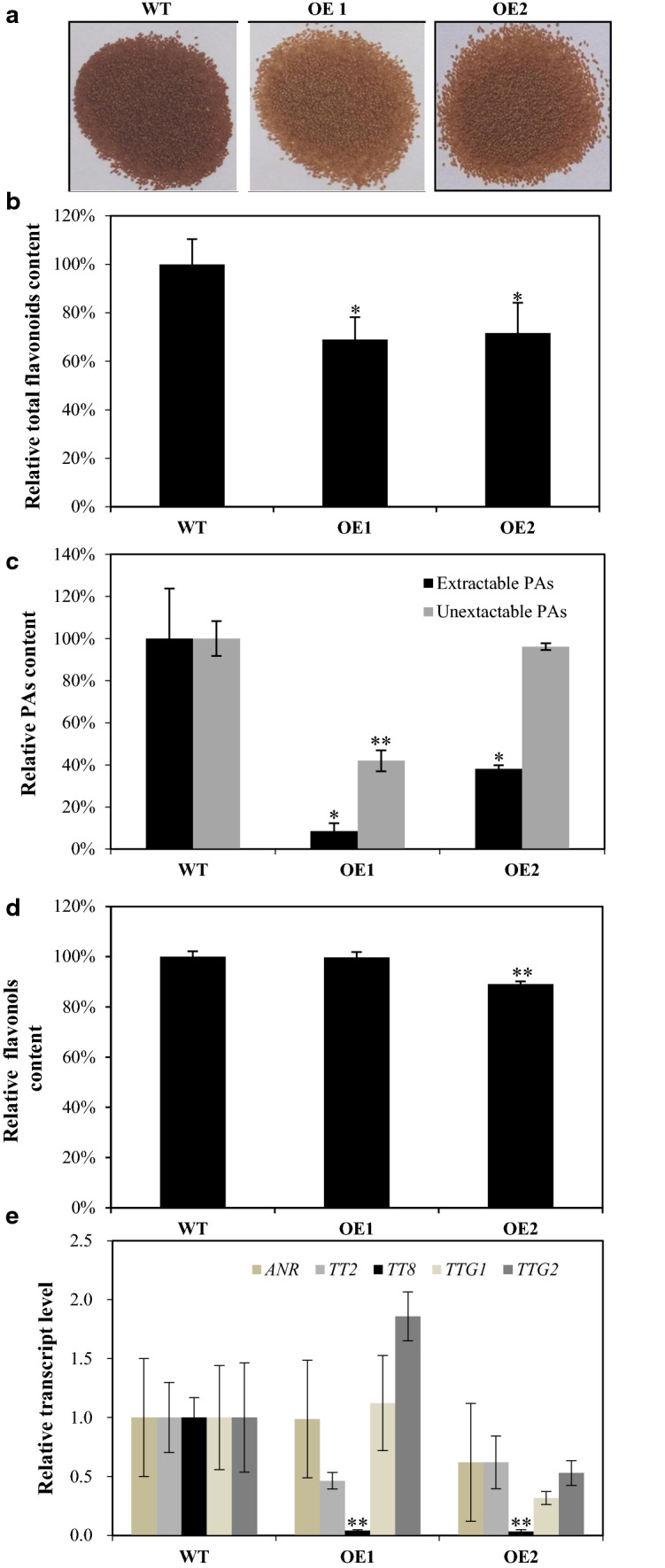
Proanthocyanidin accumulation in the seeds of transgenic *Arabidopsis* over-expressing *GbMYBR1* and the wild type. **a** Phenotypes of the seeds of the transgenic *Arabidopsis* (OE1 and OE2) compared with the wild type. **b** The relative total flavonoid contents in the seeds of the transgenic and the wild-type lines. The value in the wild type was set as 100%. **c** The relative extractable and unextractable proanthocyanidin contents in the seeds of the transgenic and the wild-type lines. The values in the wild type were set as 100%, respectively. **d** The relative flavonol contents in the seeds of the transgenic and the wild-type lines. The value in the wild type was set as 100%. **e** The relative transcript levels of genes involved in proanthocyanidins sub-pathway as determined by qPCR, and the values in the wild type were set as 1.0, respectively. Data in **b**–**e** are presented as mean ± SD, Student’s *t *test (*n* = 3, *P < 0.05, **P < 0.01)

Seeds of transgenic lines displayed light-brown coloration relative to the dark brown coloration of wild-type seeds (Fig. 6a), suggesting a decrease in proanthocyanidin and/or flavonol content. We thus measured total flavonoid content, and found that it was significantly reduced by approximately 30% in the two transgenic lines relative to the wild type (Fig. 6b).

In addition, the extractable proanthocyanidin content was significantly reduced in the two OE lines, and the unextractable proanthocyanidin content was reduced by about 50% in line OE1 as compared to the wild type (Fig. 6c). However, the flavonol profiles and total flavonol content were not affected in the transgenic line OE1 (Fig. S4a), although individual flavonol compounds changed differently (Figs. S4b, 6d). Collectively, the reduction in total flavonoid content in seeds of the transgenic lines was most likely due to the decrease of proanthocyanidins.

To further investigate the transcription changes of genes in the proanthocyanidin and flavonol biosynthetic pathways, the relative transcript levels of several key pathway genes were determined by qPCR in developing seeds (4-d-old siliques). Among all genes detected (e.g., *ANR*, *TT2*, *TT8*, *TTG1*, and *TTG2*), only the transcript level of *TT8* showed a significant reduction (Fig. 6e), indicating that the decrease of proanthocyanidins most likely resulted from the down-regulation of *TT8*. TT8 is the key bHLH protein in the MBW protein complex that regulates proanthocyanidin biosynthesis in the seeds of *Arabidopsis* (Gonzalez et al. 2009).

### GbMYBR1 physically interacts with GL3 in yeast two-hybrid assays

**Fig. 7:**
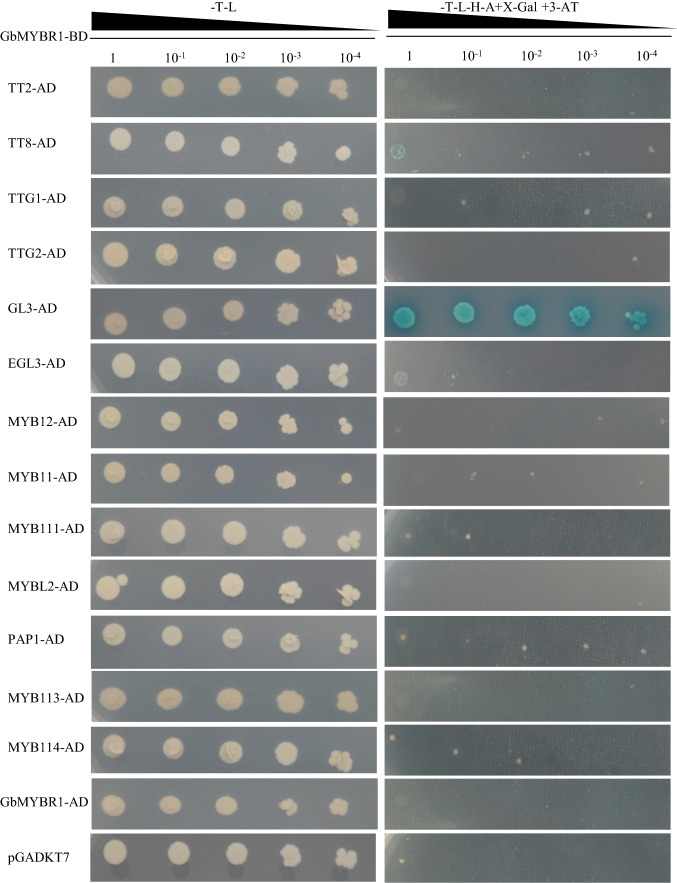
Yeast two-hybrid assay of GbMYBR1 with other transcription factors involved in flavonoid biosynthesis and trichome development. GbMYBR1 was fused with the binding domain and active domain, and the other transcript factors were fused with the active domain. Yeast cells grew on the selective medium SD-Trp-Leu, and SD-Trp-Leu-His-Ade containing X-gal and 3-AT. The yeast cells harboring GbMYBR1-BD and pGADKT7 were used as negative controls

To identify potential proteins that may interact with GbMYBR1, yeast two-hybrid assays were performed with several TFs from related pathways. The bait protein, GbMYBR1, was co-transformed with each individual prey protein, including TT2, TT8, and TTG1 from the proanthocyanidin pathway, TTG2, GL3, and EGL3 involved in trichome development, MYB12, MYB11, MYB111, and MYBL2 in the flavonol pathway, and PAP1, MYB113s, and MYB114 from the anthocyanin pathway (Fig. 7). The yeast cells harboring GbMYBR1 and other individual proteins grew well on selective medium (SD-Trp-Leu), indicating that they were successfully transformed.

However, only the yeast cells harboring GbMYBR1 and AtGL3 displayed distinct blue coloration on selective medium (SD-Trp-Leu-His-Ade) containing 3-AT (3-amino-1, 2, 4-triazole) and X-gal (Fig. 7), indicating that GbMYBR1 can physically interact with AtGL3, which is involved in the regulation of flavonoid accumulation and trichome development (Xu et al. 2014b).

### Global transcriptional analysis of *Arabidopsis* over-expressing *GbMYBR1* by transcriptome sequencing

To identify additional genes down-regulated by *GbMYBR1*, total RNAs from seedlings of the transgenic (OE1) and wild-type *Arabidopsis* were subjected to transcriptome sequencing analysis. In total, 1385 genes were down-regulated and 860 genes were up-regulated by more than 1.5-fold in the OE1 line in comparison to the wild type, which supports the idea that GbMYBR1 acts as a repressor. The gene ontology classification of the down-regulated genes is summarized in Figure S5, and it revealed that over-expression of *GbMYBR1* affected genes in multiple pathways, and particularly in the phenylpropanoid pathway.

Among the down-regulated genes, a number of lignin and flavonoid pathway genes were down-regulated at various levels, including *PAL*, *4CL*, and *CAD* genes in the lignin pathway, and *F3H, FLS*, and several *UGT* genes in the flavonoid pathway (Table 1). In particular, *ANS*, *TT19*, *PAP1*, and *PAP2* were down-regulated by more than fivefold, which was consistent with the significant reduction of anthocyanins (Fig. 4a).

**Table 1:** Summary of flavonoid pathway genes that were down-regulated by more than 1.5-fold in the *GbMYBR1* transgenic line than in the wild-type *Arabidopsis*

Gene ID	Fold change (WT/OE1)	*P* value	Gene name
AT5G17220	37.6	0.00085	Glutathione S-transferase (TT19)
AT4G22880	33.9	5.00E-05	Anthocyanidin synthase (ANS)
AT5G61160	18.8	5.00E-05	Anthocyanin 5-aromatic acyltransferase 1
AT4G15280	13.7	0.0079	UDP-glucosyl transferase 71B5
AT1G07260	12.5	5.00E-05	UDP-glucosyl transferase 71C3
AT1G66390	9.1	5.00E-05	Production of anthocyanin pigment 2 protein (PAP2)
AT1G33030	8.5	0.0006	O-methyltransferase family protein
AT4G14090	6.4	5.00E-05	UDP-Glycosyltransferase superfamily protein
AT1G56650	5.7	5.00E-05	Production of anthocyanin pigment 1 (PAP1)
AT3G21230	5.6	5.00E-05	4-coumarate:CoA ligase 5 (4CL)
AT3G21240	4.1	5.00E-05	4-coumarate:CoA ligase 2 (4CL)
AT1G08250	3.7	5.00E-05	Arogenate dehydratase 6 (ADT6)
AT1G22640	3.4	5.00E-05	Myb domain protein 3
AT3G24503	3.1	5.00E-05	Aldehyde dehydrogenase 2C4 (ALDH)
AT5G48930	2.9	5.00E-05	Hydroxycinnamoyl transferase (HCT)
AT4G34230	2.8	5.00E-05	Cinnamyl alcohol dehydrogenase 5 (CAD)
AT2G22330	2.5	5.00E-05	Cytochrome P450, family 79, subfamily B, polypeptide 3
AT4G34135	2.4	5.00E-05	UDP-glucosyltransferase 73B2
AT4G37970	2.4	0.00155	Cinnamyl alcohol dehydrogenase 6 (CAD)
AT5G04230	2.3	5.00E-05	Phenylalanine ammonia-lyase 3 (PAL)
AT4G38620	2.3	5.00E-05	Myb domain protein 4
AT3G50740	2.2	5.00E-05	UDP-glucosyl transferase 72E1
AT1G51680	2.2	5.00E-05	4-Coumarate:CoA ligase 1 (4CL)
AT5G17050	2.1	5.00E-05	UDP-glucosyltransferase 78D2
AT2G37040	2.1	5.00E-05	Phenylalanine ammonia-lyase 1
AT4G36220	2.1	5.00E-05	Ferulic acid 5-hydroxylase 1 (F5H)
AT1G78270	1.9	5.00E-05	UDP-glucosyltransferase 85A4
AT3G51240	1.9	5.00E-05	Flavanone 3-hydroxylase (F3H)
AT5G63580	1.8	0.0003	Flavonol synthase 2 (FLS)
AT3G53260	1.8	5.00E-05	Phenylalanine ammonia-lyase 2 (PAL)
AT4G30210	1.8	5.00E-05	P450 reductase 2
AT1G01420	1.6	0.00635	UDP-glucosyltransferase 72B3

Interestingly, a great number of genes associated with plant defense were also significantly down-regulated (> eightfold), including genes encoding pathogenesis-related proteins (AT4G33720 and AT3G04720), plant defensins (AT5G44430 and AT5G44420), chitinase family proteins (AT2G43580 and AT2G43590), lectins (AT3G16450 and AT3G16530), and WRKY75 (AT5G13080) (Table S3). Thus, it is possible that GbMYBR1 also has the potential to down-regulate defense-associated genes, and it is reasonable to speculate that transgenic *Arabidopsis* might be susceptible to pathogen infection.

To test this assumption, we challenged both transgenic (line OE1) and wild-type *Arabidopsis* plants with bacterium *Pseudomonas syringae* pv. tomato DC3000 (*Pst* DC3000) and *Botrytis cinerea*, as they are commonly used for pathogen infection assays in *Arabidopsis*. It was revealed that the transgenic line appeared to be more susceptible to these two pathogens, and developed larger lesions and stronger disease symptoms than did the wild-type plants (Fig. S6a, right panels). Even in the mock control treatments, the OE1 line exhibited stronger disease symptoms than the wild type (Fig. S6a, upper panels). The hydrogen peroxide levels were also higher in the OE1 line compared to the wild type, as indicated by the red pigmentation (Fig. S6b), indicating that the transgenic plants were more susceptible to *Pst* DC3000 and *Botrytis cinerea* than wild type.

## Discussion

Phenylpropanoid compounds play important roles in various growth and developmental processes in plants. The biosynthesis of phenylpropanoids is subject to sophisticated regulation by different types of TFs (Broun 2005). Among them, MYB repressors have been found to be involved in different pathways in various plants (Ma and Constabel 2019). Here, we characterized a novel R2R3-MYB repressor in *Ginkgo*, *GbMYBR1*, and revealed that it was involved in multiple processes, including plant growth, accumulation of phenylpropanoids, and trichome development. *GbMYBR1* is the first *MYB* repressor gene described in gymnosperm that exhibits pleiotropic effects on plant growth and development. Our genetic, molecular, and metabolic data demonstrated that *GbMYBR1* is distinct in many ways from several known MYB repressor genes identified in other plant species.

### GbMYBR1 possesses distinct sequence characteristics

Sequence alignment revealed that GbMYBR1 showed relatively low identity with other characterized MYB4 repressors from *Arabidopsis*, poplar, switchgrass, and grape (Fig. 1a). In particular, typical C1, C2, ZF, and C4 motifs that are diagnostic for MYB4 TFs are not present in the C-terminus of the deduced GbMYBR1 protein. The LxLxL-type EAR motif and the TLLLFR motif that are common in R2R3-MYB4-type repressors (Ma and Constabel 2019) were also lacking in GbMYBR1. However, GbMYBR1 still retained the conserved bHLH-binding motif in the R3 domain, and thus likely binds to bHLH TF(s) as part of the multiple MBW regulatory complex.

Phylogenetic analysis also suggested that GbMYBR1 could be a distinct MYB that is different from other repressors in the flavonoid pathway, which generated a specific group in the phylogenetic tree (Fig. 1b). GbMYBR1 was closely related to MYB5 from *Arabidopsis* (Li et al. 2009), FaMYB1 from *Fragaria* × *ananasa* (Aharoni et al. 2001), PtMYB182 from *Populus* (Yoshida et al. 2015), and MybC2-L1 from *Vitis vinifera* (Huang et al. 2014) that are involved in multiple processes, although GbMYBR1 showed even lower identity with these MYB5-type TFs than with MYB4-type TFs. This might be related to the special evolutional status of *Ginkgo*, which is evolutionarily distinct from other MYB4- or MYB5-type R2R3 MYB repressors.

### *GbMYBR1* regulates multiple processes

Our data from over-expression analyses in *Arabidopsis* clearly indicated that multiple processes are affected by the over-expression of *GbMYBR1*. However, GbMYBR1 is different from many other endogenous MYB4- or MYB5-type repressors that act in multiple processes in *Arabidopsis*.

The growth of the *GbMYBR1*-over-expressing *Arabidopsis* was retarded, which may be due to the reduction of lignin content, or due to the significant reduction of flavonols, in particular K3R7R. K3R7R is an endogenous inhibitor of polar auxin transport in *Arabidopsis* shoots (Yin et al. 2014). The absence of *UGT78D2*, a kaempferol-3-*O*-glycosyltransferase, disrupted K3R7R biosynthesis and led to plant stunting (Yin et al. 2014). Similarly, the stunting of the transgenic *Arabidopsis* is consistent with the decreased expression level of *UGT78D2* gene (Table 1) as well as the decrease of K3R7R content (Fig. S2). Among the *MYB4* homology genes expressed in *Arabidopsis*, over-expression of *ZmMYB42* and *EgMYB1* significantly affected *Arabidopsis* growth (Legay et al. 2010; Sonbol et al. 2009), but over-expression of *CmMYB1* (Zhu et al. 2013) and *PtMYB182* (Yoshida et al. 2015) did not, indicating that MYB4-type repressors have variable effects on plant growth when over-expressed in *Arabidopsis*.

Anthocyanins and proanthocyanidins are also among the flavonoids that were affected by *GbMYBR1* over-expression in the transgenic *Arabidopsis* plants (Figs. 4 and 6). Similarly, when a *VvMYB4*-like gene from grape was over-expressed in *Arabidopsis*, anthocyanins were greatly reduced (Perez-Diaz et al. 2016). This phenomenon is different from the function of *Arabidopsis MYB5*, in which proanthocyanidins in the *myb5* mutant was reduced, but the anthocyanin level was not affected (Li et al. 1996). However, for the other MYB repressors in *Medicago truncatula*, *MtMYB2* affected both anthocyanin and proanthocyanidin accumulation in *Arabidopsis* (Jun et al. 2015).

*Ginkgo* lacks trichome on their leaves; meanwhile, trichome density was greatly reduced in the *GbMYBR1*-over-expressing *Arabidopsis* (Fig. 5c). It was shown that several trichome-related genes including *GL2, GL3*, *TTG1*, and *TTG2* were down-regulated in the transgenic *Arabidopsis* plants (Fig. 5c), and that GbMYBR1 could bind with AtGL3 as shown through yeast-two-hybrid experiment (Fig. 7). Therefore, the trichome density could be changed by two factors: one is that the down-regulation of trichome-related genes (e.g., GL3) and the other is that these bHLH TFs are redirected by GbMYBR1 and lost their function. Nevertheless, these results clearly indicated that GbMYBR1 is a key player in the development of leaf epidermis in *Ginkgo*. However, the production of seed coat mucilage and root hairs were not affected (Fig. S3), which is different from what was observed for *Arabidopsis MYB5*. The over-expression of *AtMYB5* affected trichome development, seed coat mucilage, and root hairs (Li et al. 2009). Although flavonoids, trichome initiation, mucilage production, and root hair initiation were believed to be linked (Broun 2005), it is not the same case as for *GbMYBR1* regulation. From these phenotypic comparisons, it was clear that *GbMYBR1* is different from these MYB4- and MYB5-type repressors.

In addition, *GbMYBR1* is different from another repressor in *Ginkgo*, *GbMYBF2*, which was also over-expressed in *Arabidopsis*. *GbMYBF2* regulates flavonol and anthocyanin biosynthesis in young *Arabidopsis* seedlings by down-regulating *CHS*, *FLS*, *F3H*, and *ANS* genes, but whether it also affected plant growth or trichome development was not reported (Xu et al. 2014a). *GbMYBF2* was highly expressed in *Ginkgo* roots (Xu et al. 2014a), which is different from the expression patterns of *GbMYBR1* that was preferentially expressed in leaves (Fig. 2), suggesting that these two suppressors function in different tissues in *Ginkgo*.

### Regulatory mechanism of GbMYBR1 in *Arabidopsis*

**Fig. 8:**
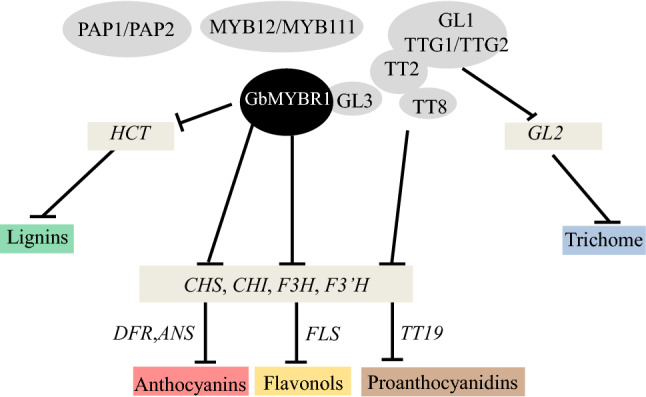
The proposed working model of GbMYBR1 involved in the phenylpropanoid pathway and trichome development in *Arabidopsis*. GbMYBR1 interacts with GL3 to compete the MBW (R2R3-MYB/bHLH/WD40) complex leading to diverse sub-pathway including lignin, flavonols, anthocyanins, proanthocyanidins, and trichome development

We found that GbMYBR1 inhibits multiple processes in different ways, and the model presented in Fig. 8 summarizes our results and details on the regulatory mechanism of *GbMYBR1* in *Arabidopsis*.

We propose that GbMYBR1 forms a complex with the bHLH cofactor GL3 to regulate multiple processes (Fig. 7). In *Arabidopsis*, GL3 is a key component of the MBW regulatory complex that regulates flavonoid biosynthesis and trichome development (Zimmermann et al. 2004). The interaction of GL3 with GbMYBR1 competes with the interaction of GL3 with MYB activators including PAP1/PAP2 in the anthocyanin pathway, TT2 in the proanthocyanidin pathway, MYB12/MYB11 in the flavonol pathway, and GL1/TTG1/TTG2 during trichome initiation. This inhibition, therefore, suppresses the expression of the corresponding downstream pathway genes (e.g., *F3H*, *DFR*, *ANS*, and *ANR*; Figs. 4, 5, 6). The repression of GbMYBR1 is clearly required for interaction with the bHLH cofactor GL3, which is consistent with the presence of the bHLH-binding motif in the R3 domain of GbMYBR1 (Fig. 1a).

Similar results were found in petunia. PhMYB27 interacts directly with both the GL3 and TT8 in yeast three-hybrid assays, although with different affinities (Albert et al. 2014). In poplar, yeast two-hybrid assays also confirmed the competition between the MYB activators and repressors (Ma et al. 2018). In *Arabidopsis*, MYB4 interacts with the bHLH cofactors GL3, TT8, and EGL3 for suppression (Wang et al. 2020), and the interaction of MYB with bHLH cofactors is not as specific as for GbMYBR1. It is most likely that GbMYBR1 functions as an active transcriptional repressor. Therefore, the replacement of one of the R2R3 MYB partners in the MBW complex with R2R3 transforms the complex from an activator to a repressor (Albert et al. 2014).

As for the monolignol biosynthetic pathway, the relevant lignin MYB activators do not require bHLH cofactors or form an MBW complex. Therefore, the lignin-type R2R3-MYB repressors appear to act primarily by binding the promoter region directly (Ma et al. 2018). This might be the same case for GbMYBR1 in the lignin pathway, as the key lignin pathway gene *HCT* was highly down-regulated (Fig. 3d). In comparison, in other studies of MYB4 repressors, when lignification was reduced, multiple genes were affected. Such examples include the over-expression of *ZmMYB42* (*COMT*, *PAL*, and *4CL)*, *EgMYB1* (*CAD* and *CCR*), *CmMYB1* (*C4H*, *4CL*, *C3H*, *CCoAOMT*, *CCR*, *F5H*, *COMT*, and *CAD*) in *Arabidopsis* (Sonbol et al. 2009; Legay et al. 2010; Zhu et al. 2013). Therefore, the suppression of GbMYBR1 on lignin is more specific than for the other MYB repressors in *Arabidopsis*.

Many investigations demonstrated that the EAR motif and/or the TLLLFR motif were essential for the proper functioning of several R2R3-MYB repressors like PtMYB182, and/or MtMYB2 (Mellway et al. 2009; Shen et al. 2012). In our study, we found that GbMYBR1 did not contain these two key motifs, indicating that EAR and/or TLLFR motifs are not essential for the proper functioning of GbMYBR1. Therefore, GbMYBR1 appears to be a distinct R2R3-MYB repressor, which is different from the AtMYB4-type (with EAR motif) or the PtMYB82-type (with both EAR and TLLLFR motifs) that were defined previously (Ma et al. 2018). Thus, GbMYBR1 is a new type of R2R3 repressor, which is evolutionarily unique to gymnosperm plants. Even though, when BLASTp was performed on the NCBI website, one putative PLN03212 repressive domain was found, which is provisionally for transcription repressor MYB5 with an interval of only 4-130aa and a very low e-value of 3.69e-55 (Lu et al. 2020), but the function of this domain has not been reported. Therefore, the functional domains of GbMYBR1-like repressors require further identification and characterization.

Overall, GbMYBR1 caused broad impacts on the regulation of phenylpropanoid biosynthesis and trichome development in *Arabidopsis*. Whether its function in biotic susceptibility is associated with lignin reduction, other flavonoids, or additional factors, GbMYBR1 needs to be further characterized. In addition, whether GbMYBR1 directly regulates pathway genes in *Ginkgo* itself, including those enlisting *HCT*, *F3H*, *DFR*, or *ANS*, still needs to be further investigated.

#### *Author contributions statement*

Yongzhen Pang designed all the experiments and perfected the final article; Xiaojia Su conducted the experiments and data analysis, and wrote the first draft of the manuscript; Yaying Xia conducted the yeast two-hybrid and pathogen infection assay experiments; Wenbo Jiang provided the expression vector; Guoan Shen provided analytical tools and performed bioinformatics analysis. All authors have read and approved the final manuscript.

## Electronic supplementary material

Below is the link to the electronic supplementary material.

Supplementary file 1 (DOCX 8538 kb)

## Abbreviations

ANS: Anthocyanin synthase
bHLH: Basic helix-loop-helix
DFR: Dihydroflavonol reductase
EGL3: Enhancer of Glabra 3
F3H: Flavanone 3-hydroxylase
FLS: Flavonol synthase
GL1: Glabrous 1
GL3: Glabrous 3
HCT: Hydroxycinnamoyl transferase
K3R7R: Kaempferol 3-*O*-rhamnoside-7-*O*-rhamnoside
MBW: MYB-bHLH-WD40 protein complex
TFs: Transcription factors
TT: Transparent testa
TTG1: Transparent testa glabra 1
TTG2: Transparent testa glabra 2
