# Supplementary material for: GbMYBR1 from Ginkgo biloba represses phenylpropanoid biosynthesis and trichome development in Arabidopsis
Source: Planta. 2020 Sep 29;252(4):68. doi: 10.1007/s00425-020-03476-1 (PMC7524859; doi:10.1007/s00425-020-03476-1)
Supplement: Supplementary file 1 — (DOCX 8538 kb) [file 425_2020_3476_MOESM1_ESM.docx]

**Supporting Material**

**Table S1.** Primer sequences used in this study

| **Primers** | **Sequence (5’-3’, the corresponding restriction sites were underlined)** | **Note** |
| --- | --- | --- |
| GbMYBR1-F | CACCATGGGTCGCTCTCCAATGTGTTC | For gene cloning |
| GbMYBR1-R | TCATGTATTCATCCAACGATAC | For gene cloning |
| GbMYBR1-RT-F | GCTCCTGCAAATCCTATGATT | For RT-PCR |
| AtGL1-RT-F | CTCCACCGTCATTGTTCATCACT | For RT-PCR |
| AtGL1-RT-R | GAGCCAGTTCTTGTTTTGGTTTATC | For RT-PCR |
| AtGL2-RT-F | ATGTCAATGGCCGTCGACATG | For RT-PCR |
| AtGL2-RT-R | TCTCGCAGCTTCTCTAGTTCCG | For RT-PCR |
| AtPAL1-RT-F | CAACGTACCCGTTGATTCAG | For RT-PCR |
| AtPAL1-RT-R | TCCTCGAAAGCTCCAATCTT | For RT-PCR |
| At4CL1-RT-F | TCAACCCGGTGAGATTTGTA | For RT-PCR |
| At4CL1-RT-R | TCGTCATCGATCAATCCAAT | For RT-PCR |
| AtHCT-RT-F | CTCTTTCCAAAGCCCTTGTC | For RT-PCR |
| AtHCT-RT-R | TCAGCCACAACGAAGAGAAC | For RT-PCR |
| AtC4H-RT-F | CGGATCTAACAAAGGAAGTGC | For RT-PCR |
| AtC4H-RT-R | TCCAATGCTCGCCGTAAACAG | For RT-PCR |
| AtCCOAOMT1-RT-F | GATTGGCTACGACAACACT | For RT-PCR |
| AtCCOAOMT1-RT-R | CAACAGGGAGCATACAGAT | For RT-PCR |
| AtCHS- RT-F | CGCATCACCAACAGTGAACAC | For RT-PCR |
| AtCHS- RT-R | TCCTCCGTCAGATGCATGTG | For RT-PCR |
| AtCHI- RT-F | CATCGATCCTCTTCGCTCTC | For RT-PCR |
| AtCHI- RT-R | AGGTGACACACCGTTCTTCC | For RT-PCR |
| AtF3H- RT-F | TCAGATCGTTGAGGCTTGTG | For RT-PCR |
| AtF3H- RT-R | ATGTCGAAACGGAGCTTGTC | For RT-PCR |
| AtF3'H- RT-F | GGCACTAAGCCTCATCGAAC | For RT-PCR |
| AtF3'H- RT-R | TTGGCGTCGTGTATTTTCAA | For RT-PCR |
| AtFLS- RT-F | ACCGTCATGCGTCAATTACA | For RT-PCR |
| AtFLS- RT-R | TCAACGCATCACGCTTTAAC | For RT-PCR |
| AtMYB11- RT-F | CCCAAAAATGCCGGGCTAAAGAGATG | For RT-PCR |
| AtMYB11- RT-R | CTTCTTCGGGAGTTATGTTT | For RT-PCR |
| AtMYB12- RT-F | CACTTTGGGAAACAGGTGGTCACT | For RT-PCR |
| AtMYB12- RT-R | GTTGTGGAGTTTACGGCTGA | For RT-PCR |
| AtMYB111- RT-F | AAAGAGGAAATATTACTTCCGACGAA | For RT-PCR |
| AtMYB111- RT-R | TTGTCTGTTCTTCCTGGTAGATGTG | For RT-PCR |
| AtMYB113- RT-F | CAGATGTGTTGGGTCCAGAA | For RT-PCR |
| AtMYB113- RT-R | AATTCAGTTCTAAAGTCTCTTC | For RT-PCR |
| ATMYB114-RT-F | ATGGAGGGTTCGTCCAAAGGGTTG | For RT-PCR |
| ATMYB114-RT-R | GACATCATTAGCGGTCCGACCAGG | For RT-PCR |
| AtDFR- RT-F | AAACGTTAGCGGAGAAAGCA | For RT-PCR |
| AtDFR- RT-R | CCTCGTTCCGAGTGATAGGA | For RT-PCR |
| AtANS- RT-F | CATCGTGGGTTGGTGAATAA | For RT-PCR |
| AtANS- RT-R | GTCCGTGGAGGAAACTTAGC | For RT-PCR |
| AtANR- RT-F | GTGACCGGTCTCAAGGAAAT | For RT-PCR |
| AtANR- RT-R | ACAGCAAATGTAGCGACCAG | For RT-PCR |
| AtTT2- RT-F | ATGGGAAAGAGAGCAACTACTAGTGTG | For RT-PCR |
| AtTT2- RT-R | TCAACAAGTGAAGTCTCGGAGC | For RT-PCR |
| AtTT8- RT-F | AGACAACTCAACCAGCGGAG | For RT-PCR |
| AtTT8- RT-R | CGTCAAATCCTCCGGTGACA | For RT-PCR |
| AtGL3- RT-F | CTGCTTAACCGTCAATTGCAAGCA | For RT-PCR |
| AtGL3- RT-R | CAACAGATCCATGCAACCCTTTGA | For RT-PCR |
| AtEGL3-RT-F | CACTAGACGAGCTTCCGCCG | For RT-PCR |
| AtEGL3-RT-R | AGTACCACTCGGTGTCGGTGAG | For RT-PCR |
| AtTTG1- RT-F | TGGCTACGATTTTGATGGATTCT | For RT-PCR |
| AtTTG1- RT-R | GATGTCTTTCAAGCTCAGCAACA | For RT-PCR |
| AtTTG2- RT-F | CCCCACAACTTTCTAAGCAAACA | For RT-PCR |
| AtTTG2- RT-R | TGCTTAGGAAGTTGTGAGTGAAG | For RT-PCR |
| AtPAP1- RT-F | ACCTCGATCCTTCACAGTTAACAAC | For RT-PCR |
| AtPAP1- RT-R | GCATGGAGGATTAACGTCAACTT | For RT-PCR |
| AtPP2A- RT-F | TATCGGATGACGATTCTTCGTGCAG | For RT-PCR |
| AtPP2A- RT-R | GCTTGGTCGACTATCGGAATGAGAG | For RT-PCR |
| AtUBQ10- RT-F | AGGTACAGCGAGAGAAAGTAGCA | For RT-PCR |
| AtUBQ10- RT-R | TAGGCATAGCGGCGAGGCGT | For RT-PCR |

**Table S2.** Genbank accession numbers referred in this study

| **Gene name** | **Accession numbers** | **Species** |
| --- | --- | --- |
| AmMYB330 | P81395.1 | *Antirrhinum majus* |
| AtMYB3 | AEC06531.1 | *Arabidopsis thaliana* |
| AtMYB32 | NP_195225 | *A. thaliana* |
| AtMYB4 | AY519615 | *A. thaliana* |
| AtMYB5 | AAC49311.1 | *A. thaliana* |
| AtMYB6 | NP_192684 | *A. thaliana* |
| AtMYB7 | BAA21618.1 | *A. thaliana* |
| CmMYB4 | AKP06188.1 | *Chrysanthemum morifolium* |
| EgMYB1 | CAE09058.1 | *Eucalyptus gunnii* |
| FaMYB1 | AAK84064.1 | *Fragaria ananassa* |
| GbMYBR1 | MH136603 | *Ginkgo biloba* |
| HvMYB1 | P20026.1 | *Hordeum vulgare* |
| PhMYB4 | ADX33331 | *Petunia hybrida* |
| PtMYB182 | XP_002305872.1 | *P. trichocarpa* |
| PvMYB4a | AEM17348.1 | *Panicum virgatum* |
| SmMYB39 | AGS55356.1 | *Salvia miltiorrhiza* |
| TaMYB1 | NP_001159044.1 | *Zea mays* |
| VvMYB4a | NP_001268129.1 | *Vitis vinifera* |
| VvMYBC2-L1 | JX050227 | *V. vinifera* |
| Zm38 | P20025.1 | *Z. mays* |
| ZmMYB31 | NP_001105949.1 | *Z. mays* |
| ZmMYB42 | NP_001106009.1 | *Z. mays* |

**Table S3.** Summary of genes that were down-regulated by more than eight fold in the *GbMYBR1* transgenic line than in the wild type *Arabidopsis*

| **Gene ID** | **Value for OE1** | **Value for WT** | **Fold change (WT/OE)** | ***P* value** | **Gene annotation** |
| --- | --- | --- | --- | --- | --- |
| **AT4G33720** | **0.0** | **21.1** |  | **5.00E-05** | **Pathogenesis-related 1 superfamily protein** |
| **AT2G16367** | **0.0** | **11.2** |  | **5.00E-05** | **Encodes a defensin-like (DEFL) family protein** |
| AT2G41240 | 0.0 | 5.8 |  | 5.00E-05 | Basic helix-loop-helix protein 100 |
| **AT4G10265** | **0.0** | **2.5** |  | **5.00E-05** | **Wound-responsive family protein** |
| AT1G11785 | 0.0 | 2.4 |  | 0.0001 | Unknown protein |
| AT5G42445 | 0.0 | 2.1 |  | 0.0044 | 60S ribosomal protein L3 (RPL3C) |
| AT1G14120 | 0.0 | 1.8 |  | 5.00E-05 | 2-oxoglutarate (2OG) and Fe(II)-dependent oxygenase superfamily protein |
| AT4G17215 | 0.0 | 1.8 |  | 5.00E-05 | Pollen Ole e 1 allergen and extensin family protein |
| AT2G43440 | 0.0 | 1.7 |  | 5.00E-05 | F-box and associated interaction domains-containing protein |
| AT2G04032 | 0.0 | 1.7 |  | 5.00E-05 | Zinc transporter 7 precursor |
| **AT5G44430** | **4.6** | **10016.5** | **2175.2** | **5.00E-05** | **plant defensin 1.2C** |
| **AT5G44420** | **9.8** | **11829.1** | **1209.6** | **5.00E-05** | **plant defensin 1.2** |
| **AT2G39030** | **1.8** | **496.3** | **277.9** | **5.00E-05** | **Acyl-CoA N-acyltransferases (NAT) superfamily protein** |
| **AT4G11650** | **0.4** | **101.2** | **264.2** | **0.00055** | **Osmotin 34** |
| **AT2G43580** | **0.5** | **88.4** | **169.8** | **0.00015** | **Chitinase family protein** |
| AT3G12502 | 1.9 | 214.7 | 113.2 | 5.00E-05 | Other RNA |
| **AT3G12500** | **2.5** | **249.3** | **100.6** | **5.00E-05** | **Basic chitinase** |
| AT3G49620 | 1.1 | 111.2 | 100.5 | 5.00E-05 | 2-oxoglutarate (2OG) and Fe(II)-dependent oxygenase superfamily protein |
| **AT4G16260** | **13.6** | **1139.3** | **83.8** | **5.00E-05** | **Glycosyl hydrolase superfamily protein** |
| **AT2G43590** | **4.1** | **240.6** | **59.1** | **5.00E-05** | **Chitinase family protein** |
| AT1G66700 | 0.3 | 17.5 | 50.6 | 0.0003 | S-adenosyl-L-methionine-dependent methyltransferases superfamily protein |
| AT3G23550 | 2.3 | 105.7 | 45.1 | 5.00E-05 | MATE efflux family protein |
| AT5G17220 | 0.6 | 23.4 | 37.6 | 0.00085 | Glutathione S-transferase phi 12 |
| AT4G22880 | 0.5 | 15.5 | 33.9 | 5.00E-05 | Leucoanthocyanidin dioxygenase |
| **AT3G04720** | **156.1** | **5105.3** | **32.7** | **5.00E-05** | **Pathogenesis-related 4** |
| AT1G26390 | 0.2 | 4.9 | 32.6 | 0.0047 | FAD-binding Berberine family protein |
| AT4G37410 | 1.2 | 35.7 | 29.6 | 5.00E-05 | Cytochrome P450, family 81, subfamily F, polypeptide 4 |
| AT3G28220 | 3.9 | 101.2 | 25.8 | 5.00E-05 | TRAF-like family protein |
| AT3G14680 | 0.2 | 5.9 | 23.9 | 0.00025 | Cytochrome P450, family 72, subfamily A, polypeptide 14 |
| **AT3G15356** | **23.1** | **525.6** | **22.7** | **5.00E-05** | **Legume lectin family protein** |
| AT2G30770 | 0.7 | 15.7 | 21.8 | 5.00E-05 | Cytochrome P450, family 71, subfamily A, polypeptide 13 |
| AT5G61160 | 11.3 | 211.9 | 18.8 | 5.00E-05 | Anthocyanin 5-aromatic acyltransferase 1 |
| **AT1G15520** | **0.4** | **7.0** | **18.1** | **5.00E-05** | **Pleiotropic drug resistance 12** |
| **AT5G13080** | **0.4** | **6.9** | **18.1** | **0.00785** | **WRKY DNA-binding protein 75** |
| **AT1G73330** | **6.1** | **109.0** | **18.0** | **5.00E-05** | **Drought-repressed 4** |
| AT4G24340 | 0.3 | 5.4 | 17.2 | 0.0015 | Phosphorylase superfamily protein |
| **AT5G67080** | **0.5** | **8.2** | **16.6** | **0.0001** | **Mitogen-activated protein kinase kinase kinase 19** |
| AT5G09670 | 1.3 | 22.1 | 16.5 | 5.00E-05 | Loricrin-related |
| AT1G47395 | 5.1 | 83.1 | 16.4 | 5.00E-05 | Unknown protein |
| **AT3G16450** | **0.8** | **12.6** | **15.9** | **5.00E-05** | **Mannose-binding lectin superfamily protein** |
| **AT4G11290** | **1.1** | **16.8** | **15.5** | **5.00E-05** | **Peroxidase superfamily protein** |
| AT1G64360 | 22.9 | 352.6 | 15.4 | 5.00E-05 | Unknown protein |
| AT1G22890 | 5.0 | 75.2 | 15.0 | 5.00E-05 | Unknown protein |
| **AT1G73260** | **5.3** | **80.1** | **15.0** | **5.00E-05** | **kunitz trypsin inhibitor 1** |
| AT1G65890 | 0.3 | 4.7 | 14.4 | 5.00E-05 | Zcyl activating enzyme 12 |
| AT5G19230 | 0.9 | 12.3 | 14.0 | 5.00E-05 | Glycoprotein membrane precursor GPI-anchored |
| AT4G15280 | 0.2 | 2.7 | 13.7 | 0.0079 | UDP-glucosyl transferase 71B5 |
| AT1G06160 | 8.5 | 113.6 | 13.3 | 5.00E-05 | Octadecanoid-responsive Arabidopsis AP2/ERF 59 |
| AT2G43150 | 15.9 | 209.5 | 13.2 | 5.00E-05 | Proline-rich extensin-like family protein |
| AT4G29690 | 0.2 | 2.2 | 12.6 | 0.008 | Alkaline-phosphatase-like family protein |
| AT1G07260 | 1.8 | 22.9 | 12.5 | 5.00E-05 | UDP-glucosyl transferase 71C3 |
| AT5G23850 | 1.1 | 13.1 | 12.4 | 5.00E-05 | Arabidopsis thaliana protein of unknown function (DUF821) |
| **AT3G49110** | **10.5** | **125.4** | **11.9** | **5.00E-05** | **Peroxidase CA** |
| AT5G44480 | 0.5 | 5.7 | 11.8 | 5.00E-05 | NAD(P)-binding Rossmann-fold superfamily protein |
| AT2G32487 | 2.4 | 27.8 | 11.8 | 5.00E-05 | Unknown protein |
| AT2G28210 | 0.5 | 5.4 | 11.3 | 0.00725 | Alpha carbonic anhydrase 2 |
| AT4G24350 | 25.3 | 275.3 | 10.9 | 5.00E-05 | Phosphorylase superfamily protein |
| **AT4G06746** | **6.2** | **67.2** | **10.8** | **5.00E-05** | **Related to AP2 9** |
| AT2G39310 | 1.7 | 17.9 | 10.7 | 5.00E-05 | Jacalin-related lectin 22 |
| AT1G47400 | 4.7 | 49.5 | 10.5 | 5.00E-05 | Unknown protein |
| AT1G26380 | 0.5 | 5.2 | 10.5 | 5.00E-05 | FAD-binding Berberine family protein |
| AT2G04460.1 | 0.7 | 7.2 | 10.4 | 5.00E-05 | Transposable element gene |
| AT1G23850 | 1.3 | 13.8 | 10.3 | 5.00E-05 | Unknown protein |
| AT3G49780 | 3.7 | 36.9 | 10.1 | 5.00E-05 | Phytosulfokine 4 precursor |
| AT3G27250 | 0.6 | 5.6 | 10.0 | 0.0002 | Unknown protein |
| AT5G40330 | 0.4 | 4.3 | 9.8 | 0.0028 | Myb domain protein 23 |
| AT4G15210 | 6.0 | 58.0 | 9.7 | 5.00E-05 | Beta-amylase 5 |
| AT3G16430 | 0.2 | 1.5 | 9.6 | 0.00845 | Jacalin-related lectin 31 |
| AT3G53980 | 6.7 | 63.6 | 9.5 | 5.00E-05 | Bifunctional inhibitor/lipid-transfer protein/seed storage 2S albumin superfamily protein |
| **AT3G25760** | **15.8** | **147.6** | **9.3** | **5.00E-05** | **Allene oxide cyclase 1** |
| **AT3G16530** | **54.5** | **504.8** | **9.3** | **5.00E-05** | **Legume lectin family protein** |
| AT1G19670 | 64.0 | 582.8 | 9.1 | 5.00E-05 | Chlorophyllase 1 |
| AT1G66390 | 1.9 | 17.5 | 9.1 | 5.00E-05 | Production of anthocyanin pigment 2 protein (PAP2) |
| AT5G25110 | 0.7 | 6.0 | 9.0 | 5.00E-05 | CBL-interacting protein kinase 25 |
| **AT2G43000** | **0.4** | **4.0** | **8.8** | **0.00375** | **NAC domain containing protein 42** |
| AT2G44480 | 0.4 | 3.4 | 8.8 | 5.00E-05 | Beta glucosidase 17 |
| AT5G10520 | 1.8 | 15.9 | 8.7 | 5.00E-05 | ROP binding protein kinases 1 |
| AT4G01390 | 3.9 | 33.9 | 8.7 | 5.00E-05 | TRAF-like family protein |
| AT1G10070 | 3.9 | 33.5 | 8.6 | 5.00E-05 | Branched-chain amino acid transaminase 2 |
| AT1G33030 | 0.6 | 5.0 | 8.5 | 0.0006 | O-methyltransferase family protein |
| AT3G10120 | 3.1 | 26.5 | 8.4 | 5.00E-05 | Unknown protein |
| **AT3G09260** | **4.8** | **40.0** | **8.3** | **0.00025** | **Glycosyl hydrolase superfamily protein** |
| **AT4G08870** | **17.6** | **145.7** | **8.3** | **5.00E-05** | **Arginase/deacetylase superfamily protein** |
| AT3G44510 | 0.3 | 2.9 | 8.2 | 0.00155 | Alpha/beta-Hydrolases superfamily protein |
| AT2G36792 | 0.6 | 4.8 | 8.1 | 0.00035 | Other RNA |
| AT2G29165 | 0.4 | 3.3 | 8.0 | 5.00E-05 | Transposable element gene |

Note：The genes that were associated with pathogen resistance were in bold.

**Fig. S1**  Relative transcript levels of *GbMYBR1* and flavonoid content in *G. biloba*. Relative expression level of *GbMYBR1* and total flavonoid content in *G. biloba* leaves at different seasons. The total flavonoids were quantified and previously reported as in Su et al., (2017). Relative expression level of *GbMYBR1* were detected by qRT-PCR. Values show the means and standard deviations of analytical triplicates.

**Fig. S2** Analyses of flavonoids profiles in the seedlings of transgenic *A. thaliana* over-expressing *GbMYBR1*. HPLC chromatograms of flavonoid profiles in seedlings of the wild type control (WT) and transgenic lines (OE1 and OE2). Representative flavonoids were labeled with arrowheads.

**Fig. S3** Morphology changes of the *GbMYBR1* over-expression *Arabidopsis* and the wild type plant. **a** The trichomes on the rosette leaves of 12-d-old seedlings for the wild type (WT) and two transgenic lines (OE1 and OE2). **b** The root hair for the wild type (WT) and two transgenic lines (OE1 and OE2). **c** The mucilage coloration in the mature seeds for the wild type (WT) and two transgenic lines (OE1 and OE2). The mucilage was stained by 0.05% ruthenium red for 48 h for observation.

**Fig. S4** Analyses of flavonoids in the mature seeds of transgenic *A. thaliana* over-expressing *GbMYBR1*. **a** HPLC chromatograms of flavonol profiles in seeds of the wild type control (WT) and transgenic lines (OE1 and OE2). **b** Relative flavonol content in seeds of the wild type control (WT) and transgenic lines (OE1 and OE2).

**Fig. S5** MapMan visualization of changes in expression levels of genes in the seedlings of the wild type in comparison with the transgenic *Arabidopsis* over-expressing *GbMYBR1*. Blue denotes up-regulation and red denotes down-regulation. Intense blue or red denotes fold changes of 1.5-fold or more.

**Fig. S6** Infection assays of the transgenic *Arabidopsis* inoculated with pathogens. **a** Phenotype of the wild type and transgenic *Arabidopsis* inoculated with bacterium *Pseudomonas syringae* pv. tomato DC3000 (*Pst* DC3000) (upper panels) and *Botrytis cinerea* (lower panels). **b** Hydrogen peroxide accumulation of the wild type and transgenic *Arabidopsis* leaves inoculated with bacterium *Pseudomonas syringae* pv. tomato DC3000 (*Pst* DC3000) (upper panels) and *Botrytis cinerea* (lower panels).
